# Nurses’ roles in the management of chronic inflammatory arthritis: a systematic review

**DOI:** 10.1007/s00296-018-4135-9

**Published:** 2018-08-20

**Authors:** Lion Vivienne, Schirmer Michael

**Affiliations:** Department of Internal Medicine, Clinic II, Medical University of Innsbruck, Anichstrasse 35, 6020 Innsbruck, Austria

**Keywords:** Public health, Organization and administration, Supply and distribution, Nursing, Nurse clinicians

## Abstract

In 2011 EULAR first published recommendations for the potential role of nurses in the management of patients with rheumatic diseases. To perform a literature update for the role of nurses in the management of chronic inflammatory arthritis (CIA) from 2010 to 2018. A systematic literature review (SLR) was performed according to the PRISMA guidelines, in accordance with the search strategies and eligibility criteria of the EULAR taskforce. The eligibility criteria were “inflammatory arthritis”, “interventions undertaken by nurses” and “relevant outcomes to answer the research questions”. Exclusion criteria were in itself contradictory outcomes, insufficient data, consideration if they did not clearly distinguish between nurses and health professionals or focused on chronic other than rheumatic diseases. Systematic reviews were classified as descriptive and excluded. Quality of selected trials was determined according to Oxford—levels of evidence 2009. A total of 48 articles and 10 abstracts were identified fulfilling the eligibility and exclusion criteria. Recommendation 1 has been well established in Europe so far. New evidence strengthens the recommendation 3, and—at least in part—recommendation 6. High evidence strengthens recommendation 4, especially for outpatients with low and stable disease activity. Some new evidence also exists for recommendations 7 and 8. This SLR reveals new evidence for the role of nurses in managing CIA patients since 2010, especially for RA-patients with low disease activity or in remission.

**Electronic supplementary material:**

The online version of this article (10.1007/s00296-018-4135-9) contains supplementary material, which is available to authorized users.

## Introduction

In 2014, the World Forum on Rheumatic and Musculoskeletal Diseases identified “worldwide and regional shortfalls in the provision of rheumatologists”, ranging from an estimated 0.5–3.8 rheumatologists per 100,000 inhabitants in Europe, compared to 0.07–3.09 in the Americas and 0.01–0.22 in Asia [1]. Especially in the countries with deficits of qualified rheumatologists, nurses already have an important role in the follow-up and treatment of patients with chronic inflammatory arthritis (CIA), especially cooperate support rheumatologists’ work. In order to define recommendations for the nurses’ role, the European League Against Rheumatism (EULAR) searched for evidence supporting standards of care and already in 2011 published European recommendations for the potential role of nurses in the management of patients with chronic inflammatory arthritis [2]. Indeed, the reduced care offered to patients with rheumatoid arthritis (RA) after moving to a nursing home can be considered as an alarming sign for health care planners [3, 4]. EULAR searched for standards of care provided by nurses across the countries for patients with chronic inflammatory arthritis and formulated minimum standards of care [5].

Since then, the EULAR recommendations were well disseminated and positively evaluated both across Europe and the United States (US) [5]. In an online survey, nurses, rheumatologists and patients highly agreed with them but application varied and was lowest in southern, eastern and central Europe. Differences across the countries and the need for further standardisation and research are evident.

The objective of this systematic review was to perform a literature search from 2010 to 2018 on the role of nurses in the management of chronic inflammatory arthritis as a follow-up of the 2011 EULAR recommendations using the PRISMA 2009 checklist (supplementary table 1), to evaluate the new trials according to the guidelines of the Oxford Centre for Evidence-based Medicine 2009.

## Methods

A systematic review was performed according to the PRISMA guidelines (supplementary table 1) [6]. The EULAR recommendations [2] were considered as protocol for this work concerning information sources, search terms and eligibility criteria.

### Search strategy: information sources and eligibility criteria

Chronic inflammatory arthritis (CIA) was defined as rheumatoid arthritis (RA), ankylosing spondylitis (AS) and psoriatic arthritis (PsA). The search items are listed in supplementary table 2, mainly including “inflammatory arthritis” and “nurse”. MEDLINE was scanned from 01/01/2010 to 01/07/2018, additional search was performed in Cochrane CENTRAL (via OVID SP Search), EMBASE (here via STN), Cumulative Index to Nursing and Allied Health Literature (CINAHL) and Psych Info (via EBSCO host search) available from 01/01/2010 to 18/09/2016.

### Article selection

The eligibility criteria were “inflammatory arthritis”, “interventions undertaken by nurses” and “relevant outcomes to answer the research questions” (with details outlined in supplementary table 2). No additional assumptions and simplifications were made. Duplicates were sorted out, including those already considered by the EULAR task force. Articles which did not fulfil the inclusion criteria had contradictory outcomes in itself or insufficient data were excluded. Articles were also considered as insufficient without abstract or study protocols published without data. Articles were not taken into consideration if they did not clearly distinguish between nurses and health professionals or focused on chronic other than rheumatic diseases. Systematic reviews were classified as descriptive and excluded. Prior to exclusion, they were checked for included articles which could be taken into consideration. Only abstracts from the past 2 years were taken into consideration, if evidence was high or concept was interesting for future research.

Both, titles and abstracts of the articles were screened by VL (Vivienne Lion) for fulfilling the eligibility criteria; their relevance was discussed with MS (Michael Schirmer). As the meaning of titles could be misleading, titles and abstracts were screened in one step.

### Data collection process

Data were extracted from reports by VL with subsequent review and discussion with MS. Literature references were collected using Mendeley Desktop (Elsevier Incorporated, New York, New York, USA). Studies were characterised by country of origin, to allow referrals to different health care systems.

### Assessment of literature quality and risk of bias

Quality of additional evidence was assessed according to the Oxford Centre for Evidence-based Medicine 2009. Identified abstracts which were not excluded by the eligibility criteria were not considered for any evidence category, as data were not fully available. Risk of bias was considered in trials on pharmaceuticals and medical devices, which were supported by the producing companies. There was no need to assess statistical methods as part of the meta-analysis. No risk of bias was searched for, like financial or personal bias.

## Results

### Study selection and characteristics

**Fig. 1:**
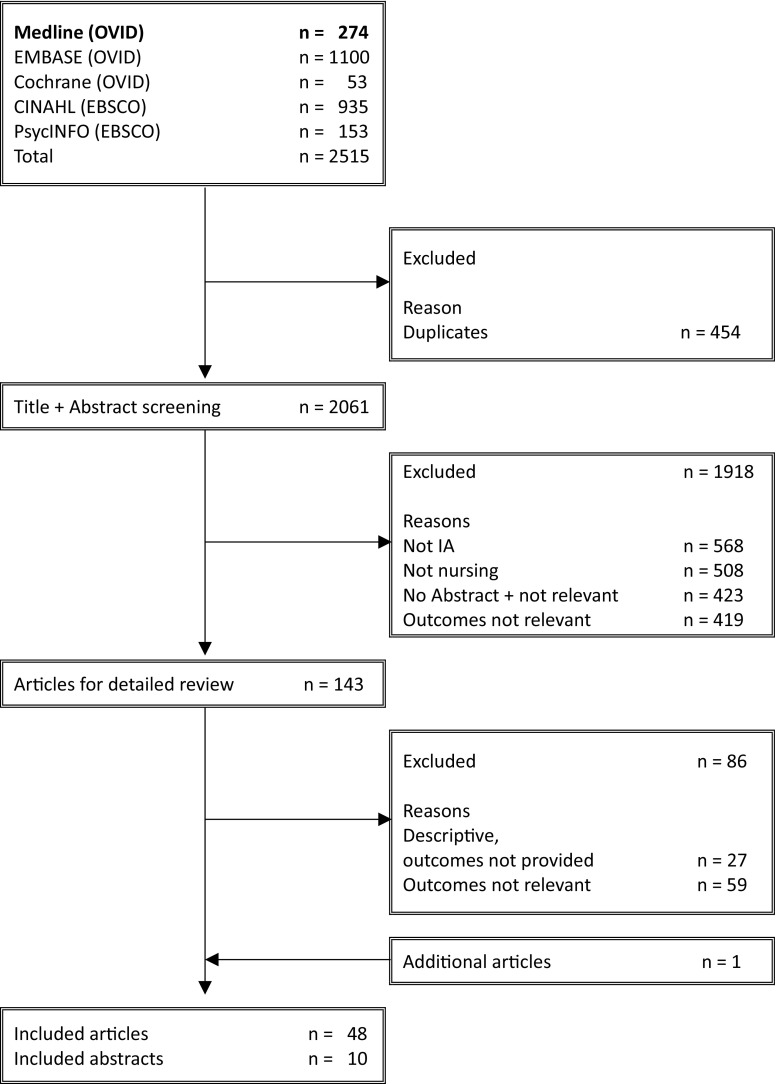
Flowchart for the results of the systematic literature review (MEDLINE (OVID) 01/01/10–01/07/18, additional searches available from 01/01/10 to 18/09/16)

A total of 2515 articles were screened, and finally 48 articles and 10 abstracts assessed as eligible and included in this review (Fig. 1): 1 meta-analysis, 17 randomised controlled trials (RCTs, summarised in Table 1), 6 quasi-experimental studies, 5 observational studies, 3 cross-sectional studies and 16 qualitative studies. Thirty-one articles exclusively dealt with the management of RA and 12 dealt with CIAs including RA. Three studies focused on rheumatic diseases in general and two on inflammatory rheumatic diseases. None of the identified articles solely considered PsA or AS. Specialised nursing personnel participated in 36 identified studies. Categories of additional evidence are presented in Table 2. Articles with studies of low evidence and abstracts were cited in this review only when higher evidence was not available. After consideration of a possible risk of bias in trials on pharmaceuticals and medical devices, which were supported by the producing companies, none of the studies was included in this SLR (supplementary table 1). No personal risk of bias was identified across the studies.

**Table 1:** Main characteristics of included meta-analysis and randomised controlled trials with high level of evidence (meta-analysis: no. 1, short-term studies: no. 2–6, long-term studies: no. 7–17)

No.	Method			Results	Refs.
	Time [mo]	[*n*] 1 vs. 2	Intervention		
1	12–24	12 mo: 164–266 vs. 183–27124 mo 87–123 vs. 88–183 (RA, AS, PsA, undifferentiated polyarthritis)	RCTs on the efficacy of nurse-led vs. physician-led follow-up	No difference for disease activity and patient satisfaction after 12 mo, better disease activity and patient satisfaction after 24 mo for nurse-led follow-up Better self-efficacy for nurse-led follow-up after 12 and 24 mo	[14] DK
2	6	38 vs. 124 (rheumatic diseases)	Usual care ± PBL-programme, group sessions 10/year	Stronger empowerment 68% changed their lifestyles	[8] SE
3	6	463 vs. 460 (stable RA)	Nurses reported comorbidities vs. patient’s disease activity self-assessment	Nurses’: more measures taken for CVD, infections, cancer, osteoporosis Self-assessment: 17% received intensification in DMARD therapy (vs. 11%), 72% documented disease activity score in booklet, 30% discussed results with rheumatologist	[28] FR
4	4	71 vs. 70 (IA, without education)	Usual care ± group and individual nurse-led education	Better global well-being, self-efficacy and activation Less pain	[9] NO
5	6	31 vs. 36 (RA)	Educational session ± motivational interview, coaching session, calls	More days per week with 30 min physical activity Higher total self-efficacy + autonomous motivation Cave: control group with better disease activity	[10] NL
6	6	14 vs. 15 (RA, hospitalised)	Usual care ± nurse case management	Lower disability levels No differences in total health care costs	[37] TR
7	12	138 vs. 152 (RA)	Patient-initiated appointments via nurse-led telephone line vs. regular planned appointments	Patient-initiated: higher satisfaction overall + with ease of contacting nurse, accessibility, convenience Helpline enabled appointments within 14 days Telephone contacts and contact costs increased No difference in total cost of service	[24] UK
8	12	71 vs. 70 (IA, without education)	Usual care ± group and individual nurse-led education	Better global well-being	[7] NO
9	21	34 vs. 31 (IA, not uncontrolled disease activity)	Consultations led by experienced rheumatology CNSs (always the same for one patient) vs. consultations led by different medical doctors	CNS group had higher satisfaction No difference in disease activity change (both improved), patient global assessment, joint pain, fatigue, quality of life and coping	[15] NO
10	12	50 vs. 47 (CIA, bDMARDs, low + stable disease activity)	Two annual monitoring visits by rheumatologists vs. one of two replaced by special trained nurse	No difference in disease activity, pain, functional impairment + satisfaction with and confidence in obtaining rheumatology care	[16] SE
11	12		See study no. 10 above	Intervention group had lower total annual cost and less fixed costs (lower nurse consultation costs)	[39] SE
12	12	91 vs. 90(RA)	Nurse-led 30 min appointments (medical history, physical examination, pain control, prescribing medication + dosage changes, steroid injections, patient education, psychosocial support, ordering blood test/X-rays, referrals to specialists) vs. rheumatologist-led 15 min appointments	Nurse-led care had higher satisfaction after 26 weeks + was not inferior in disease activity change, pain, fatigue, duration of morning stiffness, physical functioning, anxiety and depression No difference in overall costs, but lower nurse consultations costs. More cost-effective in relation to change of disease activity	[17] UK
13	12	93 vs. 96 vs. 93 (RA, low, stable disease activity, no bDMARDs)	Educational sessions + care provided by rheumatologist vs. shared care vs. nurse-led care	Disease activity increased in all groups, nursing group with higher self-efficacy	[29] DK
14	24		Prolonged study no. 12	No difference between groups	[25] DK
15	24	97 vs. 96 vs. 94	See study no. 13 above	No difference in other health care, mean intervention and total cost Lower intervention costs in shared vs. rheumatologists’ care	[41] DK
16	12	94 vs. 88 vs. 93 (RA, low disease activity)	Follow-up every 3–4 mo either by physicians in outpatient clinics vs. tele-health by nurses vs. tele-health by rheumatologists	Nurse-led tele-health care was not inferior in disease activity change, physical functioning, quality of life and self-efficacy	[22] DK
17	12	107 vs. 107 (stable RA)	Follow-up every 3 mo either by nurse (medical history, physical exam, pts education, psycho-social support, ordering blood test/X-rays, referrals to specialists) vs follow-up by rheumatologist	Nurse-led care had greater reduction in disease activity, pain, fatigue and morning stiffness Overall costs for nurse-led care were lower	[42] CN

*No*. Number, *mo* months, *n* number, *pts* patients, *vs* versus, *RA* rheumatoid arthritis, *AS* ankylosing spondylitis, *PsA* psoriatic arthritis, *RCTs* randomised controlled trials, *DK* Denmark, *PBL* problem-based learning programme, *SE* Sweden, *CVD* cardiovascular diseases, *DMARD* disease modifying antirheumatic drugs, *FR* France, *IA* inflammatory arthritis, *NO* Norway, *min* minutes, *NL* The Netherlands, *TR* Turkey, *UK* United Kingdom, *CNS* clinical nurse specialist, *CIA* chronic inflammatory arthritis, *bDMARD* biological disease modifying antirheumatic drugs, *CN* China

**Table 2:** Additional evidence of 2010–2018 literature for recommendations of rheumatology nursing management in CIA according to Oxford – levels of evidence 2009

EULAR recommendations 2011		Category of evidence	Category of additional evidence of 2010–2018 literature
1	Patients should have access to a nurse for education to improve knowledge of CIA and its management throughout the course of their disease	1B	1B
2	Patients should have access to nurse consultations in order to experience improved communication, continuity and satisfaction with care	1B	1A for satisfaction with care 4 for improved communication and continuity
3	Patients should have access to nurse-led telephone services to enhance continuity of care and to provide ongoing support	3	1B
4	Nurses should participate in comprehensive disease management to control disease activity, to reduce symptoms and to improve patient-preferred outcome	1A	1A
5	Nurses should identify, assess and address psychosocial issues to minimise the chance of patients’ anxiety and depression	1B	1B
6	Nurses should promote self-management skills in order that patients might achieve a greater sense of control, self-efficacy and empowerment	3	1A for self-efficacy 2B for empowerment Sense of control not studied
7	Nurses should provide care that is based on protocols and guidelines according to national and local contexts	3	2B
8	Nurses should have access to and undertake continuous education in order to improve and maintain knowledge and skills	3	2B
9	Nurses should be encouraged to undertake extended roles after specialised training and according to national regulations	3	3
10	Nurses should carry out interventions and monitoring as part of comprehensive disease management in order to achieve cost savings	1B	1B

### Synthesis of results

*Recommendation 1* “Patients should have access to a nurse for education to improve knowledge of CIA and its management throughout the course of their disease”

Four RCTs reported benefits for patients: several trials aimed at improving not only patients’ knowledge [7] but also patients’ self-care ability [8] and self-efficacy [9, 10], global well-being [7, 9], empowerment [8], beliefs and behaviours to manage chronic illness [9]. In addition, education resulted in trained patients showing increased physical activity [10], able to reliably determine their disease activity [11] and being more probable to quit smoking [12]. In Europe, such education provided by non-physician health professionals is well established in 24 of 27 countries [13].

*Recommendation 2* “Patients should have access to nurse consultations in order to experience improved communication, continuity and satisfaction with care”

A recent meta-analysis did not find a difference between nurse-led and physician-led follow-up after 1 and 2 years, even with low-evidence favour of nurse-led follow-up in patients with low disease-activity [14]. Direct comparisons in RCTs resulted in divergent priorities for nurses or physicians’ consultations [15–17]. After 1 year, satisfaction was equally estimated by CIA-patients with low disease activity under treatment with biologic agents, if physician-led care was replaced by a rheumatology nurse every second visit and nurses’ service of 30 min was preferred to physicians’ service of 15 min after 26 weeks but not after 52 weeks [17]. Qualitative studies confirmed the recommendation concerning improved communication [18–20] and continuity [19, 21] as experienced by the patients.

*Recommendation 3* “Patients should have access to nurse-led telephone services to enhance continuity of care and to provide ongoing support”

According to a recent RCT, an outcome-based tele-health follow-up for tight control of RA patients with low disease activity or remission can achieve similar disease control as conventional outpatient follow-up by rheumatologists [22]. Already earlier, one RCT and one quasi-experimental study showed that telephone services increase patients’; empowerment [23] and satisfaction,[23, 24] enhance their motivation [19] and ensure safety under treatment. Besides, nurse-led telephone services have been established to provide additional care and as such are incorporated in different interventional services at least in 15 of 27 European countries [13]. Telephone services may also make access to care easier, but may also be initiated by the nurse.

*Recommendation 4* “Nurses should participate in comprehensive disease management to control disease activity, to reduce symptoms and to improve patient-preferred outcomes”

Many studies including an RCT further investigated clinical outcomes using disease activity scores [7, 9, 15–17, 25–29]. Overall outcome of nurse-led care was not inferior to rheumatologists’ care as measured by disease activity in patients with low disease activity or remission, and maybe replaced even by outcome-based tele-health follow-up by nurses [22].

*Recommendation 5* “Nurses should identify, assess and address psychosocial issues to minimise the chance of patients’ anxiety and depression”

In two RCTs, nurses’ interventions did not minimise patients’ anxiety and depression [7, 9]. Nurse-led education may improve global well-being but not necessarily patients’ psychosocial health [7, 9]. According to a survey, 74% of CIA patients preferred psychological support provided by a nurse compared to 55% by a physician [30].

*Recommendation 6* “Nurses should promote self-management skills in order that patients might achieve a greater sense of control, self-efficacy and empowerment”

New RCTs studied different self-management skills (e.g. promoting education and physical exercise) and further confirmed that nurse-led interventions lead to higher self-efficacy [9, 10, 29] and more empowerment [23], especially among RA-patients, as confirmed for self-efficacy by a recent meta-analysis [14]. Sense of control was not studied.

*Recommendation 7* “Nurses should provide care that is based on protocols and guidelines according to national and local contexts”

Guidelines and protocols most often referred to medical treatment and guide in monitoring visits during treatment with biological agents. Nurse practitioners benefited very strongly from an educational programme to further improve the management of RA [31, 32].

*Recommendation 8* “Nurses should have access to and undertake continuous education to improve and maintain knowledge and skills”

Indeed, after various training programmes, nurses took over new roles [33–37] or improved their performance in the management of patients [31, 32, 38]. They performed joint examinations [33, 34] and examined gait, arms, legs and spine to distinguish between RA or non-RA [35]. Overall their work profile changed, as they gained more independence, took a more specific medical history, supported studies, provided information on infusions and administered those [36].

*Recommendation 9* “Nurses should be encouraged to undertake extended roles after specialised training and according to national regulations”

Many RCTs with participation of specialised personnel were identified. Extended roles of nurse care include consultant role, advanced clinical tasks, administration of intra-articular injections and managing patient advice lines [13]. Legal constraints may limit the wide-spread performance of these roles. For the professionals themselves, specialised training led to higher work satisfaction, more independent work and implementation of new tasks [36], and increased self-confidence, knowledge and career opportunities for the nurses [13].

*Recommendation 10* “Nurses should carry out interventions and monitoring as part of comprehensive disease management in order to achieve cost savings”

Cost reductions were reported together with stable outcome parameters in monitoring CIA-outpatients with stable and low disease activity under treatment with biological agents, with a nurse taking over every second visit of the rheumatologist [39]. Authors from different European countries performed economic analyses of various forms of nurse-led care, and the majority of economic analyses investigated an established nurse-led model of care [17, 26, 39, 40] provided by specialised nurses [17, 39–41]. Although nurse consultation costs were lower than physicians’ costs, there was only few evidence that nurse-led care decreases total costs compared to physician-led care [39]. When including loss of productivity into total costs, costs of nurse-led community care may be even higher than hospital care [40]. Cost-effectiveness studies have not been considered for this SLR, if disease activity outcome parameters were not comparable [42].

## Discussion

Taken together, important new evidence for the role of nurses in the management of CIA came up during the past years (outlined in Table 2), especially for recommendations 3 and 6. There was no contradictory evidence to any of the recommendations. Therefore, as additional provision of care to patients with rheumatic diseases will be needed in the future, nurses will be able to support rheumatologists, especially in CIA patients with stable disease and low disease activity.

Based on current evidence, the professional role of nurses will certainly change, especially in out-patient clinics. Depending on the recommendation addressed, however, the quality of new evidence widely differs. For recommendations 1 and 3 it appears that more evidence from randomised clinical trials will hardly change the clinical practice in the future, as in many European rheumatologic services patients have already access to nurses [13] and nurse-led telephone services not only in trials but also in routine clinical settings [13, 19, 43–46]. In fact, outcome-based tele-health services by nurses may even replace nurse-led visits in RA patients with low disease activity or in remission [22]. As a next step, evidence for new technical tools is growing as for mail services [24, 44, 47, 48].

The main tasks of nurses in rheumatic services, to perform a comprehensive disease management to control disease activity, to reduce symptoms and to improve patient-preferred outcomes have also been further supported by new evidence—and there is certainly more space for other responsibilities of nurses in the future, especially in the disease management of outpatients with low and stable disease activity [15, 16, 25, 29, 40]. For these tasks nurses’ support by other health care professionals may be helpful, as has been shown for example to increase physical activity [10] and to quit smoking [12].

Besides, evidence increases for nurse-led education, especially for CIA-patients with stable disease which led to improved global well-being [7, 9], self-efficacy [9, 10], self-assessment of disease activity [11], empowerment [8], activation [9] and knowledge [7]. Besides, nurses can contribute to self-injection training of medical treatment [49, 50], train disease-activity self-assessment [28], follow-up care after a self-care promoting programme [29] and self-regulation sessions together with follow-up phone calls [10]. Initiatives are ongoing to improve the quality of nurse-led education [51]. The use of an educational needs assessment tool (ENAT) like for RA in the UK [51] allows patients to indicate their educational needs at each rheumatologic visit to further increase their self-efficacy by focusing on their individual needs [52]. Translations of ENAT for RA-patients are available for nine European languages so far [52].

For the future, educational efforts for the patients will have to concentrate on long-term repetitive interactions to ensure a minimum level of patients’ knowledge during the course of the disease, sometimes even leading to change of lifestyle behaviour [8, 10]. RA- and PsA-patients trained by a rheumatologist and a health psychologist may become more independent in interpreting blood results and checking for side effects of MTX therapy themselves, resulting in up to 55% fewer visits to the clinical nurse specialist, 7% fewer visits to the rheumatologist and 39% fewer visits to the general practitioner, when nurses only provide a telephone helpline as usual care [43]. This may reduce unnecessary appointments at the rheumatology clinics and even total costs of rheumatologic care in the future—given a comparable or even better outcome. Calculations of costs with adjustments for health care quality solely based on questionnaires, however, appear critical and available data are not convincing. As a matter of fact, specific training programmes for nurses are necessary before they can take over new roles [33–37, 53] or improve their performance in the management of patients [32, 38, 54]. Guidelines and protocols have to be developed and further disseminated among professionals involved in care [54]. Besides, more studies on nurses’ care have to be performed for SpA and PsA, as the level of evidence is higher for nurses caring for RA patients than for SpA and PsA.

The most important strength of this review is that it was performed according to the PRISMA guidelines [6]. Besides, a detailed table was set up to summarize the characteristics of the included studies (Table 1).

A limitation of this review is that most studies on nurses’ care have been performed for RA patients, and the level of evidence is higher for nurses caring for RA patients than for SpA and PsA. Also, most studies focus on patients in stable and low disease activity.

Based on this SLR, future trials are needed, especially for nurse-led services to patients with diseases other than CIA, to define extended roles of nurse-led services like supporting triage efforts, implementing treat-to-target guidelines and Improving effective utilisation of care by multidisciplinary teams.

In conclusion, there is increasing evidence for the role of nurses in the management of patients with chronic inflammatory arthritis. Some recommendations are already practiced in routine clinical work, whereas RCTs are still needed for others.

## Electronic supplementary material

Below is the link to the electronic supplementary material.

Supplementary material 1 (DOCX 39 KB)

Supplementary material 2 (DOCX 88 KB)
