# Supplementary material for: Nurses’ roles in the management of chronic inflammatory arthritis: a systematic review
Source: Rheumatol Int. 2018 Aug 20;38(11):2027–36. doi: 10.1007/s00296-018-4135-9 (PMC6208652; doi:10.1007/s00296-018-4135-9)
Supplement: Supplementary file 2 — Supplementary material 2 (DOCX 88 KB) [file 296_2018_4135_MOESM2_ESM.docx]

## Supplementary table 2: Search terms for systematic literature review

OVID SP, Medline and Cochrane Central

1. exp arthritis, rheumatoid/

2. ((rheumatoid or reumatoid or revmatoid or rheumatic or reumatic or revmatic or rheumat$

or reumat$ or revmarthrit$) adj3 (arthrit$ or artrit$ or diseas$ or condition$ or nodule$)).tw.

3. (felty$ adj2 syndrome).tw.

4. (caplan$ adj2 syndrome).tw.

5. (sjogren$ adj2 syndrome).tw.

6. (sicca adj2 syndrome).tw.

7. still$ disease.tw.

8. bechterew$ disease.tw.

9. exp Spondylarthropathies/

10. (ankylos$ or spondyl$).tw.

11. (bekhterev$ or bechterew$).tw.

12. (Marie adj struempell$).tw.

13. exp Arthritis, Psoriatic/

14. (psoria$ adj (arthriti$ or arthropath$)).tw.

15. ((arthriti$ or arthropath$) adj psoria$).tw.

16. undifferentiated oligoarthritis.tw.

17. or/1-16

18. exp Inflammatory Bowel Diseases/

19. exp Arthritis/

20. 18 and 19

21. ((inflamm$ or ibd or crohn$ or enteropath$) adj5 (arthrit$ or arthrop$)).tw.

22. (granulomatous colitis adj5 (arthrit$ or arthrop$)).tw.

23. (ulcerative colitis adj5 (arthrit$ or arthrop$)).tw.

24. (granulomatous enteritis adj5 (arthrit$ or arthrop$)).tw.

25. (regional enteritis adj5 (arthrit$ or arthrop$)).tw.

26. (Ileocolitis adj5 (arthrit$ or arthrop$)).tw.

27. (terminal ileitis adj5 (arthrit$ or arthrop$)).tw.

28. (regional ileitis adj5 (arthrit$ or arthrop$)).tw.

29. or/17,20-28

30. exp Nurse/

31. (Nurs$ adj (care or role$ or skill$ or competenc$ or care or assessment$ or diagnosis or

intervention$)).tw.

32. exp nursing/

33. (registered adj nurs$).tw.

34. (qualified adj nurs$).tw.

35. (student adj nurs$).tw.

36. (practice$ adj nurs$).tw.

37. (nurs$ adj administrator).tw.

38. exp nurse practitioner/

39. (expert adj nurs$).tw.

40. (advanced adj nur$ adj pract$).tw.

41. (nurs$ adj (led or manag$ or clinic$ or directed)).tw.

42. exp nurse clinicians/

43. advanced practice nurse.mp.

44. nurse.mp.

45. nurse practi*.mp.

46. Clinical nurse specialist$.mp.

47. rheumatology nurs*.mp.

48. physician assistant$.mp.

49. (nurs$ adj (consultant$ or specialist$ or counsel$)).tw.

50. or/30-49

51. 29 AND 50

STN (EMBASE)

1. RHEUMATOID ARTHRITIS

2. ((RHEUMATOID OR REUMATOID OR REVMATOID OR RHEUMATIC OR REVMATIC OR RHEUMAT?

OR REUMAT? OR REVMARTHRIT?) 3A (ARTHRIT? OR ARTRIT? OR DISEAS? OR CONDITION? OR NODULE?))

3. (FELTY? (2A) SYNDROME)

4. (CAPLAN? (2A) SYNDROME)

5. (SJOGREN? (2A) SYNDROME)

6. (SICCA (2A) SYNDROME)

7. STILL? DISEASE

8. BECHTEREW? DISEASE

9. SPONDYLOARTHROPATHY

10. (ANKYLOS? OR SPONDYL?)

11. (BEKHTEREV? OR BECHTEREW?)

12. (MARIE (A) STRUEMPELL?)

13. PSORIATIC ARTHRITIS

14. (PSORIA? (A) (ARTHRITI? OR ARTHROPATH?))

15. ((ARTHRITI? OR ARTHROPATH?) (A) PSORIA?)

16. UNDIFFERENTIATED OLIGOARTHRITIS

17. 1 OR 2 OR 3 OR 4 OR 5 OR 6 OR 7 OR 8 OR 9 OR 10 OR 11 OR 12 OR 13 OR 14 OR 15 OR 16

18. INFLAMMATORY BOWEL DISEASE

19. ARTHRITIS

20. 18 AND 19

21. ((INFLAMM? OR IBD OR CHRON? OR ENTEROPATH?) (5A) (ARTHRIT? OR ARTHROP?))

22. (GRANULOMATOUS COLITIS (5A) (ARTHRIT? OR ARTHROP?))

23. (ULCERATIVE COLITIS (5A) (ARTHRIT? OR ARTHROP?)

24. (GRANULOMATOUS ENTERITIS (5A) (ARTHRIT? OR ARTHROP?))

25. (REGIONAL ENTERITIS (5A) (ARTHRIT? OR ARTHROP?)

26. (ILEOCOLITIS (5A) (ARTHRIT? OR ARTHROP?))

27. (TERMINAL ILEITIS (5A) (ARTHRIT? OR ARTHROP?))

28. (REGIONAL ILEITIS (5A) (ARTHRIT? OR ARTHROP?))

29. 17 OR 20 OR 21 OR 22 OR 23 OR 24 OR 25 OR 26 OR 27 OR 28

30. NURSE

31. (NURS? (A) (CARE OR ROLE? OR SKILL? OR COMPETENC? OR CARE OR ASSESSMENT? OR DIAGNOSIS OR INTERVENTION?))

32. NURSING

33. (REGISTERED (A) NURS?)

34. (QUALIFIED (A) NURS?)

35. (STUDENT (A) NURS?)

36. (PRACTICE? (A) NURS?)

37. (NURS? (A) ADMINISTRATOR)

38. NURSE PRACTITIONER

39. (EXPERT (A) NURS?)

40. (ADVANCED (A) NUR? (A) PRACT?)

41. (NURS? (A) (LED OR MANAG? OR CLINIC? OR DIRECTED))

42. NURSE CLINICIANS

43. ADVANCED PRACTICE NURSE

44. NURSE PRACTI?

45. FILE=EMBASE CLINICAL NURSE SPECIALIST?

46. RHEUMATOLOGY NURS?

47. PHYSICIAN ASSISTANT?

48. (NURS? (A) (CONSULTANT? OR SPECIALIST? OR COUNSEL?))

49. 30 OR 31 OR 32 OR 33 OR 34 OR 35 OR 36 OR 37 OR 38 OR 39 OR 40 OR 41 OR 42 OR 43 OR 44 OR 45 OR 46 OR 47 OR 48

50. 29 AND 49

51. 50 AND 2010-2016/PY

52. 51 AND PD<=20160918

53. 52 NOT MEDLINE/FS

EBSCO host search (CINAHL, PsycINFO)

CINAHL

1. (MH “arthritis, rheumatoid+”)

2. TX ((rheumatoid or reumatoid or revmatoid or rheumatic or reumatic or revmatic or

rheumat* or reumat* or revmarthrit*) and (arthrit* or artrit* or diseas* or condition* or

nodule*))

3. TX (felty* syndrome)

4. TX (caplan* syndrome)

5. TX (sjogren* syndrome)

6. TX (sicca syndrome)

7. TX (still* disease)

8. TX (bechterew* disease)

9. (MH “Spondylarthropathies+”)

10. TX (ankylos* or spondyl*)

11. TX (bekhterev* or bechterew*)

12. TX (Marie struempell*)

13. (MH “Arthritis, Psoriatic+”)

14. TX (psoria* and (arthriti* or arthropath*))

15. TX ((arthriti* or arthropath*) and psoria*)

16. TX (undifferentiated oligoarthritis)

17. (MH “Inflammatory Bowel Diseases+”)

18. (MH “Arthritis+”)

19. 17 and 18

20. TX ((inflamm* or ibd or crohn* or enteropath*) and (arthrit* or arthrop*))

21. TX (granulomatous colitis and (arthrit* or arthrop*))

22. TX (ulcerative colitis and (arthrit* or arthrop*))

23. TX (granulomatous enteritis and (arthrit* or arthrop*))

24. TX (regional enteritis and (arthrit* or arthrop*))

25. TX (Ileocolitis and (arthrit* or arthrop*))

26. TX (terminal ileitis and (arthrit* or arthrop*))

27. TX (regional ileitis and (arthrit* or arthrop*))

28. (MH “Nurses+”)

20. TX (Nurs* and (care or role* or skill* or competenc* or care or assessment* or diagnosis

or intervention*))

30. nursing

31. TX (registered nurs*)

32. TX (qualified nurs*)

33. TX (student nurs*)

34. TX (practice* nurs*)

35. TX (nurs* administrator)

36. (MH “nurse practitioners+”)

37. TX (expert nurs*)

38. TX (advanced nurs* pract*)

30. TX (nurs* and (led or manag* or clinic* or directed))

40. nurse clinicians

41. advanced practice nurse

42. (MH "Advanced Practice Nurses+")

43. nurse

44. nurse practi*

45. Clinical nurse specialist

46. rheumatology nurs*

47. physician assistant*

48. nurs* and (consultant* or specialist* or counsel*)

49. or/28-48

50. or/1-16

51. 50 or/19-27

52. 49 AND 51

PsycINFO

1. DE “Rheumatoid Arthritis”

2. TX ((rheumatoid or reumatoid or revmatoid or rheumatic or reumatic or revmatic or

rheumat* or reumat* or revmarthrit*) and (arthrit* or artrit* or diseas* or condition* or

nodule*))

3. TX (felty* syndrome)

4. TX (caplan* syndrome)

5. TX (sjogren* syndrome)

6. TX (sicca syndrome)

7. TX (still* disease)

8. TX (bechterew* disease)

9. TX (ankylos* or spondyl*)

10. TX (bekhterev* or bechterew*)

11. TX (Marie struempell*)

12. TX (psoria* and (arthriti* or arthropath*))

13. TX ((arthriti* or arthropath*) and psoria*)

14. TX (undifferentiated oligoarthritis)

15. psoriatic arthritis

16. spondylarthropathies

17. or/1-16

18. Inflammatory Bowel Diseases

19. DE “Arthritis”

20. 17 and 18

21. TX ((inflamm* or ibd or crohn* or enteropath*) and (arthrit* or arthrop*))

22. TX (granulomatous colitis and (arthrit* or arthrop*))

23. TX (ulcerative colitis and (arthrit* or arthrop*))

24. TX (granulomatous enteritis and (arthrit* or arthrop*))

25. TX (regional enteritis and (arthrit* or arthrop*))

26. TX (Ileocolitis and (arthrit* or arthrop*))

27. TX (terminal ileitis and (arthrit* or arthrop*))

28. TX (regional ileitis and (arthrit* or arthrop*))

29. or/17,20-28

30. DE "Nurses" OR DE "Psychiatric Nurses" OR DE "Public Health Service Nurses" OR DE

"School Nurses"

31. TX (Nurs* and (care or role* or skill* or competenc* or care or assessment* or diagnosis

or intervention*))

32. DE ”Nursing”

33. TX (registered nurs*)

34. TX (qualified nurs*)

35. TX (student nurs*)

36. TX (practice* nurs*)

37. TX (nurs* administrator)

38. nurse practitioner*

39. TX (expert nurs*)

40. TX (advanced nurs* pract*)

41. TX (nurs* and (led or manag* or clinic* or directed))

42. nurse clinicians

43. advanced practice nurse*

44. nurse

45. nurse practi*

46. Clinical nurse specialist

47. rheumatology nurs*

48. physician assistant*

49. nurs* and (consultant* or specialist* or counsel*)

50. or/30-49

51. 29 AND 50
